# Emergence of Toscana Virus in Europe

**DOI:** 10.3201/eid1111.050869

**Published:** 2005-11

**Authors:** Rémi N. Charrel, Pierre Gallian, José-María Navarro-Marí, Loredana Nicoletti, Anna Papa, Mária Paz Sánchez-Seco, Antonio Tenorio, Xavier de Lamballerie

**Affiliations:** * Université de la Méditerranée, Marseille, France; † Etablissement Français du Sang Alpes-Méditerranée, Marseille, France; ‡ Hospital Universitario Virgen de la Nieves, Granada, Spain; § Istituto Superiore di Sanita, Rome, Italy; ¶ Aristotle University of Thessaloniki, Thessaloniki, Greece; # Instituto de Salud Carlos III, Madrid, Spain

**Keywords:** Toscana virus, phlebovirus, emergence, Europe, meningitis, perspective

## Abstract

Toscana virus (TOSV) is an arthropodborne virus first identified in 1971 from the sandfly *Phlebotomus perniciosus* in central Italy. Many case reports in travelers and clinical research and epidemiologic studies conducted around the Mediterranean region have shown that TOSV has a tropism for the central nervous system (CNS) and is a major cause of meningitis and encephalitis in countries in which it circulates. In central Italy, TOSV is the most frequent cause of meningitis from May to October, far exceeding enteroviruses. In other northern Mediterranean countries, TOSV is among the 3 most prevalent viruses associated with meningitis during the warm seasons. Therefore, TOSV must be considered an emerging pathogen. Here, we review the epidemiology of TOSV in Europe and determine questions that should be addressed in future studies. Despite increasing evidence of its major role in medicine as an emerging cause of CNS infections, TOSV remains an unstudied pathogen, and few physicians are aware of its potential to cause CNS infections.

## Abstract 2

In southern Europe, Toscana virus is one of the three leading causes of aseptic meningitis.

Toscana virus (TOSV) was originally isolated in 1971 from the sandfly *Phlebotomus perniciosus* collected in Monte Argentario (Grosseto province, central Italy) (*1**,**2*). Thus far, most clinical and epidemiologic studies have been conducted in Italy, although studies from other Mediterranean countries have been published recently. From these, TOSV appears to be 1 of the 3 major viral pathogens involved in aseptic meningitis acquired during the summer in these countries. A bibliographic search using "Toscana virus" as keyword in the PubMed database retrieved 54 research and review articles. Less than 50% of them report imported or autochthonous human cases acquired in Italy, Spain, Portugal, France, and Cyprus. Even though evidence that TOSV plays a major role in human disease is increasing, it remains poorly studied, and physicians have little awareness of its potential to cause CNS infections.

## Virus Properties and Classification

According the 8th report of the International Committee on Taxonomy of Viruses, TOSV is a serotype of Sandfly fever Naples virus within the genus *Phlebovirus* in the family *Bunyaviridae*. TOSV is an arthropodborne virus. The lack of biochemical and genetic data for most phleboviruses dictates that the species are defined by serologic relationships and are distinguishable by 4-fold differences in 2-way neutralization tests. Phleboviruses contain a negative-sense, single-stranded RNA genome that consists of 3 segments, designated large, medium, and small, which encode the RNA-dependent RNA polymerase, the envelope glycoproteins, and the nucleoprotein, respectively.

## Epidemiology of Phleboviruses and Toscana Virus

Phlebotomus (sandfly) fever viruses have been isolated from sandflies in southern Europe, Africa, central Asia, and the Americas, and evidence exists for the presence of different viruses in the same sandfly population. Sandfly fever Naples (but not the TOSV serotype) and Sicilian viruses have the widest geographic distribution, in parallel to their vector's (*Phlebotomos papatasi*) distribution. Until recent years, the known distribution of TOSV was limited to Italy and Portugal (*3**,**4*). In Italy, the virus was isolated from the vectors *P. perniciosus* and *Phlebotomus perfiliewi* and from humans, whereas the presence of the virus in Portugal was suspected on the basis of a strain isolated from the cerebrospinal fluid (CSF) of a Swedish patient who was returning to his home country from Portugal. More recently, the geographic distribution of the virus has been extended to France, Spain, Slovenia, Greece, Cyprus, and Turkey, according to results from viral isolation and serologic surveys (*5**–**9*).

### Geographic Distribution of Toscana Virus

#### Italy

Preliminary clues pointing to the role of TOSV in CNS infections in Italy were provided by reports of imported cases diagnosed in the United States (*10*) and Germany (*11*). A large study carried out from 1977 to 1988 showed that the virus was the cause of meningitis in 2 regions of Italy, Tuscany and Marche, with a seasonal peak in August, which corresponded to the peak of sandfly activity (*3*). Since then, the virus has been isolated in other regions of central and southern Italy. More recently, research into TOSV as an etiologic agent of neurologic diseases has been carried out in Emilia-Romagna and Piedmont (*12*). Striking evidence that TOSV was the most prominent viral etiologic agent in summertime meningitis was reported in the late 1990s (*13*); in one of the most comprehensive studies, TOSV represented 81% of the viruses detected in CSF from patients who sought treatment for meningitis and other CNS infections (*14*). TOSV sequences were detected in 85 of 104 CSF specimens that provided positive results for viral sequence; However, 173 CSF specimens were negative by polymerase chain reaction (PCR); therefore, TOSV sequences were detected in 30% of the patients admitted for meningitis and in 40% of the patients admitted from June to November. A study of children living in rural or suburban areas of Siena (central Italy) showed that 40% of meningitis or encephalitis cases could be linked to TOSV infection (15). A 7-year study performed in Siena showed that 52% of aseptic meningitis cases in adults were associated with TOSV (seroconversion, presence of immunoglobulin M [IgM], PCR detection) (*16*). All studies agree regarding the monthly distribution of human cases of TOSV infections: the highest risk of acquiring TOSV is in August, then July and September, and finally June and October. Populations living in rural areas and with high levels of outdoor activity are at the greatest risk of TOSV infection. An occupational risk study conducted on forestry workers in Siena, Florence, and Arezzo showed that 77.2% of them had positive IgG for TOSV, compared with an urban population who exhibited a 22% prevalence for IgG. In contrast, 6% of forestry workers of the Piedmont area showed TOSV IgG (17). The first report of TOSV infection in Umbria was published in 2003 in the form of a retrospective study of 93 aseptic meningitis and meningoencephalitis cases. Of interest is the observed 16% of the healthy control population who were IgG positive (*12*). TOSV infections in Emilia-Romagna were documented for the first time in 2002 (*18*).

#### France

The first case of TOSV infection acquired in France was reported in a German traveler who was returning from southern France (*19*). During surveillance for West Nile virus in southern France, serum specimens from patients with suspected cases (meningitis) were tested for TOSV, and several contained specific IgM. Two cases of meningitis caused by TOSV were diagnosed by seroconversion and by viral isolation (*6*). Two cases (1 meningitis and 1 febrile illness) were recently reported (*5*). Together, data confirm that TOSV circulates in southeastern France and causes disease in humans.

#### Spain

**Figure 1:**
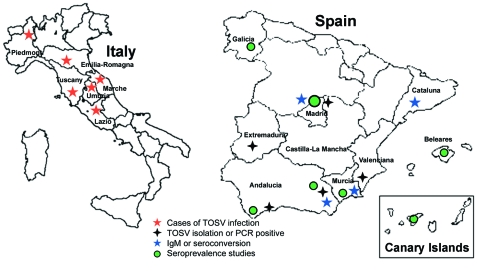
Provinces of Italy and Spain in which clinical cases of Toscana virus (TOSV) infection have been documented, and seroprevalence studies were conducted. PCR, polymerase chain reaction; IgM, immunoglobulin M.

The first case of TOSV infection reported from Spain occurred in a Swedish tourist after a visit to Catalonia and was documented by plaque reduction neutralization test (PRNT) (*20*). In the last 3 years, Spanish researchers and physicians have reported many cases and conducted large epidemiologic studies that established TOSV as 1 of the 3 leading causes of meningitis in Spain (Figure 1) (*8**,**21**,**22*). A large study conducted in different regions of Spain showed the presence of IgG antibodies to TOSV (26.2%), sandfly fever Naples virus (2,2%), and sandfly fever Sicilian virus (11.9%) in 1,181 adults and 87 children (*21*). In 2003, the EVITAR (Enfermedades Viricas Transmitidas por Artropodos y Roederes) network for the study of arthropod- and rodentborne viral diseases was created and sponsored by the Spanish Ministry of Health. Within this context, a study on seroprevalence in Granada showed a 24.9% seroprevalence rate. A significant increase was observed with age (9.4% in persons <15 years vs. 60.4% in persons >65 years). In addition, several cases of TOSV have been documented in the south, central, and Mediterranean areas. These data suggest that the situation in Spain is similar to that observed in France, with lower prevalence of CNS infections than that observed in central Italy.

#### Cyprus

Several studies were conducted in Swedish United Nations soldiers based in Cyprus in 1985. Blood samples were obtained from a 362-soldier battalion just before and immediately after their 6-month tour of duty. Of 298 serum pairs available, seroconversion to TOSV was observed in 1 patient who did not show any clinical manifestations (*9*). Seroprevalence studies showed that 20% of the healthy population had TOSV IgG (*23*).

#### Greece

Phleboviruses are found in Greece (*24*). Recent studies of populations living on the Ionian Islands and western mainland of Greece showed a seroprevalence of 60% and 35% respectively, by enzyme-linked immunosorbent assay (ELISA). However, so far, no studies have reported meningitis or encephalitis cases caused by TOSV in Greece.

#### Portugal

To date, Sweden has had 1 imported case in a man who had a severe headache and fever without neck stiffness after returning from Portugal. Viral isolation was successful and identification was performed by plaque neutralization (*4*). In addition, 1 German patient returning from vacation in Portugal had meningitis; diagnosis was established by ELISA and confirmed by immunoblot assay (*25*).

#### Germany

In a seroepidemiologic survey of 859 healthcare workers and medical students, anti-TOSV IgG was detected in 1.0% of samples by immunofluorescent assay (IFA), and in 0.7% by enzyme immunoassay (EIA). In 2,034 German patients, who were hospitalized for various diseases, 1.6% were positive for anti-TOSV IgG by IFA, and 0.8% by EIA. Anti-TOSV IgG was detected in 43 samples of commercial immunoglobulins at titers of 10–1,000 by EIA. Although the seroprevalence of antibodies to TOSV is low in Germany, TOSV infection should be considered in patients returning from virus-endemic areas who have fever and headaches or symptoms of meningitis (*26*).

### Cycle in Nature

#### Vectors

TOSV was isolated from *P*. *perniciosus* and *P*. *perfiliewi* but never from *P*. *papatasi*. TOSV has also been isolated from the brains of the bat *Pipistrellus kuhli*, which was trapped in areas where *P*. *perniciosus* and *P*. *perfiliewi* are found (*1**,**2*). Transovarial transmission has been demonstrated in the laboratory and by viral isolation from male *Phlebotomus* spp. Venereal transmission from infected males to uninfected females has also been demonstrated. *P*. *perniciosus* is distributed throughout the Mediterranean region as 2 races. The typical *P*. *perniciosus* race occurs in Italy as well as in Malta, Tunisia, and Morocco. The Iberian race replaces it in southern Spain (with the pni mtDNA sublineage) (*27*).

#### Reservoir

The reservoir of TOSV is most likely the vector. Neither mammals nor birds have been recognized as a potential reservoir, although few studies have been carried out on mammals and almost none on birds. Whether humans can play a role in the virus cycle by infecting naïve sandflies is not known.

Although a number of phleboviruses have been isolated from the blood of sick persons and from wild animals, the role of vertebrates in the maintenance of the transmission cycle of these viruses remains unclear. Transient and low-level viremia is present after phlebovirus infection in humans and in susceptible laboratory animals (*28**–**30*). Moreover, sandflies must ingest a large quantity of virus to become infected (*31*). Verani et al. (*1*) examined different species of wild vertebrates (wild mouse, bank vole, stone marten, coypus, porcupine, bat, fox, and hedgehog) through serologic testing and viral isolation.

## Disease in Humans

### Clinical Forms

Seroprevalence studies suggest that a proportion of infections by TOSV are asymptomatic or paucisymptomatic. Additional studies will be necessary to evaluate the ratio of symptomatic versus asymptomatic or paucisymptomatic infections.

In some cases, TOSV infection causes a self-limiting febrile illness without CNS manifestations; these patients are not usually hospitalized, and their cases are not usually investigated further. This fact may account for the probable underestimation of TOSV infection rates.

After an incubation period ranging from a few days to 2 weeks, disease onset is intense (70%) with headache (100%, 18 h–5 days), fever (76%–97%), nausea and vomiting (67%–88%), and myalgias (18%). Physical examination may show neck rigidity (53%–95%), Kernig signs (87%), poor levels of consciousness (12%), tremors (2.6%), paresis (1.7%), and nystagmus (5.2%) (L. Nicoletti, pers. comm.). In most cases reported so far, CSF contained >5–10 cells with normoglycorachia and normoproteinorachia. Blood samples may show leukocytosis (29%) or leukopenia (6%). The mean duration of the disease is 7 days, and the outcome is usually favorable.

Although TOSV infection in most cases consists of a mild disease with a favorable outcome, a small number of severe cases have been reported in the literature. Two young brothers and a sister living in Umbria experienced TOSV infection in the form of severe meningoencephalitis with stiff neck, deep coma, maculopapular rash, diffuse lymphadenopathy, hepatosplenomegaly, renal involvement, skin rash with lamellar desquamation, a tendency to bleed, and diffuse intravascular coagulopathy. CNS manifestations occurred after 3 weeks of fever. Convalescence was marked by hydrocephalus that required a ventriculoatrial shunt. Diagnosis was established by serologic means and by PCR sequencing (*32*). Two cases of encephalitis without meningitis were recently diagnosed by serologic testing and by detecting TOSV sequences in CSF (*33*). One case of meningitis, complicated by abducens nerve palsy, was reported (*34*). To date, no published data exist that suggest that TOSV causes any other manifestations. However, a substantial proportion of infection likely results in asymptomatic or paucisymptomatic cases (*5**,**35*).

## Laboratory Diagnosis

### Serologic Testing

Seroconversion and the detection of IgG, IgM, or both, can be achieved in cells infected with TOSV. However, cross-reactivity exists between members of the genus *Phlebovirus* and specifically between TOSV and other serotypes of sandfly fever Naples virus.

ELISAs have been developed with either crude antigens or purified virus obtained from infected cells. The advantage of ELISA resides in its capacity to rapidly test a large number of specimens; however, cross-reactions most likely will be observed. Recently, an ELISA test based on a recombinant nucleoprotein gene was developed and is now available commercially from an Italian company. Recent seroprevalence studies were based on this test (*8**,**21*).

PRNT is the test of choice when the virus species must be confirmed. Therefore, seroprevalence data must be carefully interpreted since in most cases, analyses were performed with ELISA or IFA that cannot discriminate between sandfly fever Naples virus, sandfly fever Sicilian virus, and TOSV.

### Virus Isolation

Viruses can be isolated from clinical samples by using CSF but not serum. CSF specimens that yield virus through cell culture are collected in the first 2–4 days of the disease.

TOSV replicates in a variety of animals. Intracranial, intraperitoneal, and subcutaneous routes lead to death in newborn mice, and intracranial and intraperitoneal routes lead to death in weanling mice. This effect is seen with viruses from only a few families, including flaviviruses, which are also implicated in viral encephalitis. In guinea pigs and rabbits, intracranial injection results in paralysis and death, whereas intraperitoneal injection is not fatal and results in antibody synthesis.

TOSV replicates in Vero, BHK-21, CV-1, and SW13 cells with cytopathic effect and not in C6/36 cells. However, cell culture appears to have a low sensitivity for detecting TOSV since only 14% of the PCR-positive CSF specimens added to Vero cells led to viral isolation.

### Molecular Techniques

In some cases, the relatively low level of virus in blood and CSF samples hampers attempts to isolate the virus. In such cases, molecular techniques based on PCR are more sensitive than IgM detection or viral isolation. Three different methods for molecular diagnosis of TOSV have been developed (Table). To date, all studies aimed at the molecular detection of TOSV sequences in the CSF of patients with meningitis or other CNS manifestations have used classic PCR detection through single-round or nested protocols. Tests of an RT-PCR assay alone, without a further nested PCR step, showed that this method did not appear to be valid for detecting TOSV, since no sample was positive after the first reaction. Two systems (*14**,**36*) use specific primers in the S segment, and the other is based on degenerate oligonucleotides targeting of the L segment (*37*). The most widely used has been successful for TOSV diagnosis in Italy (*13*) and France (R. Charrel et al. unpub. data). In 2003, a new method for detecting TOSV of Italian or Spanish origin was produced by using degenerate primers. The description of 2 genotypes of TOSV demonstrates a need for caution when designing molecular methods for diagnosis to avoid false-negative results. Recently, real-time PCR systems, including a fluorescent dye–labeled probe, have dramatically reduced the risk of contamination. The sensitivity of real time RT-PCR is close to that obtained by nested PCR protocols, and the results are obtained within 3 hours. However, to develop real-time PCR assays that detect all variants of TOSV circulating in Mediterranean countries and causing diseases in humans, a considerable amount of work must be done to determine the sequences of strains reflecting viral heterogeneity observed in different countries. The recent report of a Spanish genotype, genetically divergent from the strains circulating in Italy, which is not detected by PCR systems previously reported in Italy, underlines the requirement for a large program of strain isolation and full-length genome sequencing to achieve this goal.

**Table:** Primers described in the literature for TOSV virus RT-PCR and nested PCR detection*

TOSV strain	Primer	Gene	Assay	Reference
TV1	5´-CCAGAGGCCATGATGAAGAAGAT-3´	N	RT-PCR	14
TV2	5´-CCACTCCTATGAGCAGCTTCT-3´	N	RT-PCR	14
TV3	5´-AACCTGATTTCAGTCTACCAGTT-3´	N	Nested	14
TV4	5´-TTGTTCTCAGAGATGGATTTATG-3´	N	Nested	14
TosN123	5´-GAGTTTGCTTACCAAGGGTTTG-3´	N	RT-PCR	37
TosN829	5´-AATCCTAATTCCCCTAACCCCC-3´	N	RT-PCR	37
TosN234	5´-AACCTTGTCAGGGGNAACAAGCC-3´	N	Nested	37
TosN794	5´-GCCAACCTTGGCGCGATACTTC-3´	N	Nested	37
NPhlebo1+	5´-ATGGARGGITTTGTIWSICIICC-3´	L	RT-PCR	37
Nphlebo1–	5´-AARTTRCTIGWIGCYTTIARIGTIGC-3´	L	RT-PCR	37
Nphlebo2+	5´-WTICCIAAICCIYMSAARATG-3´	L	Nested	37
Nphlebo2–	5´-TCYTCYTTRTTYTTRARRTARCC-3´	L	Nested	37
ATos2–	5´-RTGRAGCTGGAAKGGIGWIG-3´	L	Nested†	37
T1	5´-CTATCAACATGTCAGACGAG-3´	N	RT-PCR	36
T2	5´-CGTGTCCTGTCAGAATCCCT-3´	N	RT-PCR	36
T3	5´-CATTGTTCAGTTGGTCAA-3´	N	Nested	36
T4	5´-CGTGTCCTGTCAGAATCCCT-3´	N	Nested	36

*TOSV, Toscana virus; RT-PCR, reverse transcription–polymerase chain reaction. †Primer used in combination with Nphlebo2+ for a nested reaction specific for TOSV.

## Genetic Diversity of TOSV Strains

### Strains Isolated

The prototype TOSV strain, ISS Phl.3, isolated from *P*. *perniciosus* in 1971 has been completely sequenced. A total of 84 virus strains were obtained from 16,374 male and female sandflies (*P*. *perniciosus* and *P*. *perfiliewi*) collected in 2 localities of the Tuscany region of Italy between 1980 and 1985. Thirty-seven (44%) were identified as TOSV and 47 (56%) as a new member of the phlebotomus fever serogroup, Arbia virus. The overall virus isolation rate from sandflies was 0.5%. Viral isolation rates for both viruses were similar in different years and in the 2 localities, suggesting that the 2 virus types were active in the sandfly population simultaneously (maximum activity in July) (*3*). Seventeen strains of TOSV have been isolated in Spain from patient specimens (*22*). Several strains have been isolated in southeastern France from patients with clinical cases and remain to be characterized.

### Genetic Diversity

A number of strains from Italy have been partially sequenced, and only minor differences in the nucleoprotein were found among strains isolated in the early 1980s from both species of sandflies, from the bat, and from humans, with <1 amino acid substitution (L. Nicoletti, pers. comm.). Similar results were described in a study on some variants in the N gene of strains isolated from humans from 1995 to 1998; only 1 variant showed a single amino acid substitution of an 80-amino-acid region (*38*). Changes in the amino acid sequence that make this protein less efficient in its interaction with the viral nucleic acid may kill the virus.

A different situation has been described in Spain for partial sequences in the large segment encoding the polymerase activity. A phylogenetic analysis performed from L segment sequences obtained from 11 clinical isolates from Granada and compared with the homologous sequence of an Italian reference strain showed that Spanish sequences were closely related to one another and distantly related to the Italian strain (*37*). This finding suggests the presence of at least 2 geographically distinct populations of TOSV.

### Phylogeny and Evolution

**Figure 2:**
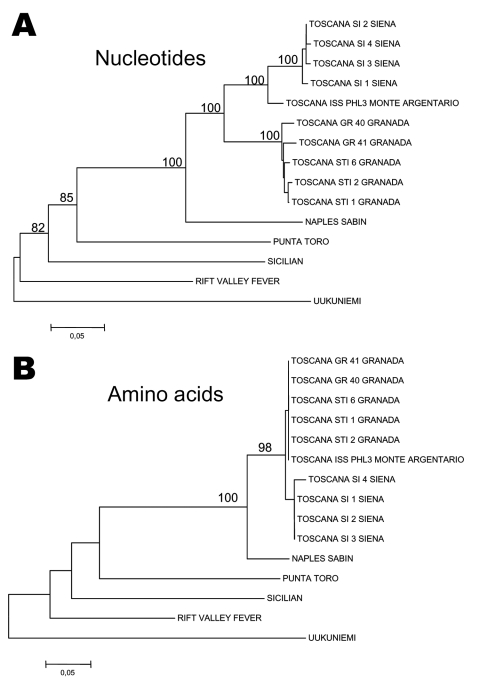
Phylogenetic trees reconstructed from nucleotide (A) and amino acid (B) sequences corresponding to a 236-nucleotide fragment of the N gene. Alignments were obtained with ClustalX 1.8 and p-distance matrices were obtained. Neighbor-joining by using 100 pseudoreplications for the bootstrap tests were carried out after excluding gaps from the alignments. Bootstrap values <75% are not shown. The numbers attached to branches are bootstrap values. A value of 0.05 substitutions per site is equivalent to 5% changes.

To date, sequence data are too scarce to perform significant phylogenetic analyses. We must therefore set up a large program of complete genome sequencing of the strains collected in different regions and simultaneously to encourage the development of viral isolation programs in all countries surrounding the Mediterranean where vectors are circulating to better understand the genetic diversity, phylogenetic relationships, and mechanisms driving the evolution of TOSV (Figure 2).

## Future Concerns

### Nature of the Vector in Different Regions

The virus has been isolated from *P*. *perfiliewi* and *P. perniciosus*, the most abundant sandfly species present in Italy. However, other vector species, found in different geographic areas, could transmit the virus. Serologic results indicate that the virus is present in many areas of the Mediterranean basin. Entomologic studies must be conducted to better understand the distribution and identification of potential vectors of TOSV.

### TOSV and Blood Donation

The recent introduction of West Nile virus into North America has stimulated a renewed interest among health authorities regarding arthropodborne viruses, specifically concerning human blood products. Until 2002, the risk of transmitting West Nile virus to a naïve patient from a blood donation was considered negligible, given the supposed short time (≈6 days) and low viremia titers. However, ≈30 cases of viral transmission were documented in 2002 and 2003 in the United States and Canada, as well as cases of West Nile virus infections after organ transplantation from a viremic donor. Moreover, 540 positive blood donation samples were detected by using PCR, which underlines the necessity of this kind of test in an epidemiologic situation similar to that seen in the United States. Recent data on TOSV circulation in Mediterranean countries during the summer raise concerns about potential implications for blood donations.

### Genotypes and Their Distribution

Limited studies have been conducted on the genetic variability of TOSV. The work of Sanchez-Seco on the L segment demonstrated the presence of 2 geographically distinct populations of the virus (*37*). However, the study was performed on strains isolated from patients with acute neurologic disease. On the basis of seroprevalence in a healthy population, Magurano and Nicoletti hypothesized that among the different strains of TOSV that may circulate in the same area and infect humans, only a few cause severe disease, whereas most strains induce antibody response with minor or no symptoms of illness (*39*). The role of different strains in the symptoms and influence on the severity of TOSV infection requires clarification.
